# Progress in Surgery

**Published:** 1901-08-03

**Authors:** 


### PROGRESS IN SURGERY.

### PERITONISM.

During the last twenty years, and more especially during the last ten, we have learnt and unlearnt a great deal about the peritoneum. The term "peritonitis" still has terrors for us, but we can at least, nowadays, see what is going on by exploratory operation: the hidden processes have become visible and even palpable to us. We know that by its rapid powers of throwing out lymph and plastic material and by its equally rapid powers of absorption it again and again shuts off and imprisons dangerous septic foci and endeavours to take into the circulation what is of detriment in its cavity. It has, in fact, its own methods of dealing with various lesions which it behoves us to try to understand and follow, not to thwart.

It is not, as a rule, a "tolerant" membrane. Occasionally cases occur in which the bowel, covered with its delicate peritoneum, has been exposed by gun-shot and other wounds to contact with all sorts of filth, and yet a simple washing has sufficed to ward off evil consequences; but as a rule it violently resents the contact of dirt of any kind, and gives warning of its injury by sudden and alarming signs. What are these signs? Let us take the rupture of an appendix or a stomach. The degree varies, but whatever the violence done to the peritoneum the symptoms are similar; pain, collapse, with weak pulse, shallow respirations, clammy, cold skin, and pinched and anxious countenance. Also vomiting. This condition of shock has been called by Gübler "peritonism." It not only occurs in acute cases where the morbid processes of peritonitis are active, but it is found occasionally in subacute cases, where post mortem, there may be no sign of peritoneal inflammation, except, perhaps, distension of the bowels.

In seeking for an explanation of this condition one naturally thinks of certain laboratory experiments. Tapping a frog's abdomen will lower or stop its heart's action. This is entirely a reflex nervous phenomenon. The afferent impulse appears to come from the peritoneum and act through the cardio-inhibitory nerves. Regnier and Richel have injected boiling water into the peritoneal cavity of the rabbit with the result that the animal died of shock within twenty-four hours; the larger the surface of peritoneum exposed to the hot water the greater was the shock. No signs of peritonitis, however, were found post mortem.

This evidence, added to that furnished by the rougher methods of disease, shows that "peritonism" may be altogether due to nerve shock. But in most cases there exists from the first, or there is soon added, another important factor, viz., distension and paralysis of the bowel. In the case of obstructions the gut contains, frequently, a large quantity of putrid fluid fæces, but sometimes the swollen bowel contains only gases of decomposition. In the latter cases the distension is important chiefly as a sign of paralysis from shock, although the mere pressure caused by the evils of inflated bowel immensely increases the discomfort and danger. But in those cases where the bowels contain fluid matter there is another danger added, viz., septic absorption and poisoning. This auto-intoxication no doubt is in itself a sufficient cause for death; and when an obstruction in the bowel is relieved it is important that this putrid matter should be removed by purgatives or in certain cases by opening and draining the bowel.

During the last few hours of life—whatever the impending cause of death may be—the mind is generally clouded and sensation impaired by some toxic matter circulating in the blood. These "death poisons" are almost always lethal in their action, and the patient passes away in an unconscious condition. But death from peritonitis is quite different from this as a rule. The faculties are clear or only partially obscured; the muscular strength in the limbs persists almost to the close, and the patient may sit up in bed, move his arms and legs freely, and even walk, a few minutes before the end, some time after the pulse in the wrist has ceased to be perceptible. Everything, in fact, points to the predominant feature of depression of the circulation and nervous shock.

Apart from the treatment of the peritoneum, the above facts clearly indicate that stimulation, by the injection of strychnine, enemata of brandy and beef tea, etc., is clearly indicated. This may stave off the fatal termination, and give time for surgical aid. This latter will vary, of course, according to the disease which produces the peritonitis; but one general measure may be briefly discussed, namely, that of irrigation and drainage. About drainage there cannot be two opinions. Experiments both in the laboratory and by disease have clearly shown that free drainage is one of the greatest safeguards against septic peritonitis. Germs may be freely introduced if there is an equally free exit for them without the terrible risk that at once follows their retention. It is impossible to overstate the importance of drainage. But irrigation has been of late strongly condemned. Why, it has been asked, should germs be spread broadcast over the peritoneum by a fluid which is too mild to destroy many of them, but which *must* diffuse them and make a general peritonitis more certain? So strong does this argument seem, and so obvious, that irrigation has been looked upon as a mistaken proceeding. But "obvious" truths are dangerous in sciences which are so obscure as pathology; and something, at least, may be said in favour of irrigation.

Wegner, in 1876, showed experimentally the enormous absorbing power of the peritoneum, amounting in one hour to as much as 8 per cent. of the bodyweight. It rapidly takes up, therefore, normal saline solution, and the osmotic current set up by the injection of this solution into the peritoneal cavity may be used as a potent means for absorbing poisonous matter. This argument has been dealt with by Dr. Clark in the *Pennsylvania Medical Bulletin* for May, 1901. He advocates free irrigation with normal saline solution rather than a "dry toilet." Then again the results do not seem to justify a definite judgment in favour of merely mopping out. More evidence is wanted before the rival claims of these two methods can be settled.

